# A Response Regulator from a Soil Metagenome Enhances Resistance to the β-Lactam Antibiotic Carbenicillin in *Escherichia coli*

**DOI:** 10.1371/journal.pone.0120094

**Published:** 2015-03-17

**Authors:** Heather K. Allen, Ran An, Jo Handelsman, Luke A. Moe

**Affiliations:** 1 Food Safety and Enteric Pathogens Research Unit, National Animal Disease Center, Agricultural Research Service, United States Department of Agriculture, Ames, Iowa, 50010, United States of America; 2 Department of Plant & Soil Sciences, University of Kentucky, Lexington, Kentucky, 40546-0312, United States of America; 3 Department of Molecular, Cellular and Developmental Biology, Yale University, New Haven, Connecticut, 06520-8103, United States of America; Dong-A University, Republic of Korea

## Abstract

Functional metagenomic analysis of soil metagenomes is a method for uncovering as-yet unidentified mechanisms for antibiotic resistance. Here we report an unconventional mode by which a response regulator derived from a soil metagenome confers resistance to the β-lactam antibiotic carbenicillin in *Escherichia coli*. A recombinant clone (βlr16) harboring a 5,169 bp DNA insert was selected from a metagenomic library previously constructed from a remote Alaskan soil. The βlr16 clone conferred specific resistance to carbenicillin, with limited increases in resistance to other tested antibiotics, including other β-lactams (penicillins and cephalosporins), rifampin, ciprofloxacin, erythromycin, chloramphenicol, nalidixic acid, fusidic acid, and gentamicin. Resistance was more pronounced at 24°C than at 37°C. Zone-of-inhibition assays suggested that the mechanism of carbenicillin resistance was not due to antibiotic inactivation. The DNA insert did not encode any genes known to confer antibiotic resistance, but did have two putative open reading frames (ORFs) that were annotated as a metallopeptidase and a two-component response regulator. Transposon mutagenesis and subcloning of the two ORFs followed by phenotypic assays showed that the response regulator gene was necessary and sufficient to confer the resistance phenotype. Quantitative reverse transcriptase PCR showed that the response regulator suppressed expression of the *ompF* porin gene, independently of the small RNA regulator *micF*, and enhanced expression of the *acrD*, *mdtA*, and *mdtB* efflux pump genes. This work demonstrates that antibiotic resistance can be achieved by the modulation of gene regulation by heterologous DNA. Functional analyses such as these can be important for making discoveries in antibiotic resistance gene biology and ecology.

## Introduction

Culture-independent metagenomic analysis of soils has led to myriad discoveries concerning the genetics, genomics, and ecology of bacteria that inhabit this environment. While most metagenomic studies of soil are based on high-throughput sequencing of total DNA or 16S rRNA gene amplicons, functional screening of metagenomic DNA libraries (functional metagenomics [1]) offers a different method for discovering genes of interest from soil metagenomes [2]. Functional metagenomics is the expression of metagenomic DNA in a host bacterium, and one of the most straightforward functional metagenomic approaches is selection for antibiotic resistance. This strategy has been applied to diverse soils with various potential for selection pressure due to anthropogenic activities [2–8]. These environments include remote soils absent of human intervention as well as soils on which antibiotics and resistant bacteria are applied, such as manure-amended soils. From these and other studies, a picture emerges of soil as a rich reservoir for antibiotic resistance genes [9]—even in so-called “pristine environments”. Furthermore, the genes recovered using functional metagenomic selection often show little homology with canonical antibiotic resistance genes that have been identified from bacteria to date, thereby illustrating the power of this approach for resistance gene discovery [2,4].

The β-lactams consistently make up greater than 50% of the antibiotic market share, with the cephalosporins and broad-spectrum penicillins the two largest classes [10], and the widespread dissemination of β-lactam resistance represents a major medical concern [11,12]. The β-lactam antibiotics inhibit bacterial d,d-transpeptidase enzymes, ultimately disrupting the periplasmic steps of peptidoglycan synthesis [13,14]. By definition, therefore, β-lactams must traverse the outer membrane of Gram negative bacteria to reach the periplasmic space. In *E*. *coli*, they do so primarily via passive diffusion through outer membrane porins [17,18], most notably OmpF and OmpC. From the periplasm, β-lactams can be pumped back across the outer membrane using one of a number of TolC-assisted pumps [15]. The AcrAB-TolC, AcrAD-TolC, and MdtABC-TolC pumps of *E*. *coli* have been implicated in increased β-lactam tolerance [15]. However, resistance to β-lactams is more commonly mediated by β-lactamase enzymes [16]. The mechanisms by which bacteria are known to develop resistance to the β-lactams therefore include decreased influx, increased efflux, target modification, and acquisition of a β-lactamase [16]. Here we describe a foreign two-component response regulator gene from an Alaskan soil metagenome that confers resistance to the β-lactam carbenicillin on *E*. *coli*.

Two-component signal transduction is a common mechanism by which bacteria sense and respond to environmental stimuli [17]. The two components comprise a sensor histidine kinase protein, which typically contains periplasmic receptor and cytoplasmic kinase/phosphatase domains, and a response regulator protein. The sensor kinase senses the stimulus, which results in the transfer of a phosphoryl group from its cytoplasmic domain to an aspartate residue on the response regulator receiver domain. Phosphorylation of the response regulator receiver domain results in activation of its output domain. An active response regulator typically achieves its goal by directly altering bacterial transcription of genes necessary for response to the original stimulus [18]. Two-component regulation is ubiquitous within the prokaryotic domain, with the numbers of two component proteins varying among bacteria from only a few to over 250 proteins (*E*. *coli* has and 29 sensor kinases and 32 response regulators) [18]. Our study shows that the introduction of a foreign response regulator gene into *E*. *coli* can directly impact gene expression, leading to an antibiotic resistance phenotype.

## Materials and Methods

### Bacterial strains, plasmids, and growth conditions

Bacterial strains and plasmids used in this study are in Table 1. The metagenomic libraries AK10, AK14, AK16, and AK18 are in the vector pCF430 [19] and are reported elsewhere [2]. Metagenomic libraries were expressed in TransforMax EPI300 *Escherichia coli* cells (Epicentre, Madison, WI, USA) for antibiotic resistance screening purposes. Metagenomic libraries were selected on eight β-lactam antibiotics at 24°C and 37°C as described [2]. The recombinant clone designated as βLR16 (Table 1) was recovered only on carbenicillin (50μg ml^-1^) after growth at 24°C. Subsequent growth of the parent clone and subclones was in Luria-Bertani (LB) medium at 24–28°C. Growth media was amended with tetracycline at 20 μg ml^-1^ or kanamycin at 20 μg ml^-1^ for plasmid maintenance where appropriate. The DNA sequence of the βLR16 DNA insert was previously submitted to GenBank (EU408353.1).

**Table 1:** Strains and plasmids used in this study.

Strain or plasmid	Genotype or relevant characteristics	Reference or Source
*E*. *coli* strains		
EPI300	*F^-^ mcrA Δ(mrr-hsdRMS-mcrBC)Φ80dlacZΔM15 ΔlacX74 recA1 endA1 araD139 Δ(ara, leu)7697 galU galK λ^-^ rpsL (Str^R^) nupG trfA dhfr*	Epicentre, Madison, WI
BL21(DE3)	*F- ompT hs*dSB(rB–, mB–) *gal dcm* (DE3)	Novagen, Madison, WI
BW25113	Host strain for Keio knockout collection; *F-* Δ*(araD-araB)567* Δ*lacZ4787*(::*rrnB*-*3*) *λ* ^-^ *rph-1* Δ*(rhaD-rhaB)568 hsdR514*	[28] Coli Genetic Stock Center (CGSC), Yale Univ.
Δ ompF	*ompF* gene deletion from Keio collection; BW25113 Δ *ompF746*::kan; Kan^R^	CGSC, Yale Univ.
Δ ompC	*ompC* gene deletion from Keio collection; BW25113 Δ *ompC768*::kan; Kan^R^	CGSC, Yale Univ.
Plasmids		
pCF430	Host vector for metagenomic library; Tet^R^	[19]
βLR16	Metagenomic clone in pCF430 conferring Crb^R^ phenotype on *E*. *coli*	[2] GenBank accession EU408353.1
pUVRA28b	Vector with native *E*. *coli* promoter driving expression from pET28b multicloning site; Kan^R^	This study
pCRC01UVRA	Subclone of βLR16 metallopeptidase gene in pUVRA28b; Kan^R^	This study
pCRC02UVRA	Subclone of βLR16 metallopeptidase and regulator genes in pUVRA28b; Kan^R^	This study
pCRC03UVRA	Subclone of βLR16 regulator gene in pUVRA28b; Kan^R^	This study

### Antibiotic susceptibility testing

The antibiotic susceptibility of βLR16 and subclones was tested in minimum inhibitory concentration assays according to CLSI guidelines except for lower incubation temperatures (24–28°C) [20]. Briefly, serial two-fold dilutions (from 512 μg ml^-1^ to 0.5 μg ml^-1^, no antibiotic in the last well) of antibiotics were made in Mueller-Hinton (MH) broth (Becton, Dickinson and Company, Sparks, MD, USA) in 96-well plates. The following antibiotics were tested: ampicillin, rifampin (both from Research Products International Corp., Mt. Prospect, IL, USA), carbenicillin (Fisher Scientific, Fair Lawn, NJ, USA), ciprofloxacin (Wako Chemicals USA, Inc., Richmond, VA), erythromycin (Fluka BioChemika, Buchs, Switzerland), amoxicillin, cefamandole, cefoxitin, ceftazidime, cephalexin, piperacillin, chloramphenicol, nalidixic acid, fusidic acid, and gentamicin (all from Sigma, St. Louis, MO, USA). Each well was inoculated with 10 μl containing 10^5^ colony forming units of each clone being tested. The assay was performed in duplicate at least three times, and *E*. *coli* strains EPI300, BL21(DE3), or BW25113 (Table 1) containing empty vector always served as the negative controls. The minimum inhibitory concentration (MIC) corresponded to the antibiotic concentration of the first well in which no growth was visible.

### Transposon mutagenesis

Plasmids were isolated by alkaline lysis followed by ethanol precipitation [21]. *In vitro* transposon mutagenesis of βLR16 was carried out with the GPS-1 Genome Priming System (New England Biolabs, Beverly, MA, USA). Insertion mutants that failed to grow on carbenicillin had presumed insertions in the active gene and were sequenced using the manufacturer’s primers. Additional insertion mutants were randomly chosen for sequencing the entire insert. All sequencing reactions were carried out at the University of Wisconsin-Madison DNA sequencing facility using Big Dye Terminator (v. 3.1, Applied Biosystems, Foster City, CA, USA). The Lasergene software package (DNASTAR, Madison, WI, USA) was used to assemble and annotate the sequence, which accesses the Basic Local Alignment Search Tool (BLAST) [22] through the National Center for Biotechnology Information (NCBI).

### β-lactam hydrolysis assay

Thick MH agar (Becton, Dickinson and Company) plates were swabbed with an overnight culture of EPI300 *E*. *coli* across each plate three times, turning the plate 60° between swabs. Then, using the wide end of a sterile P1000 pipetman tip, an agar plug was aseptically removed from the center of each plate. *E*. *coli* strain EPI300 containing pCF430, βLR16, βLR2, or no plasmid was inoculated into four separate tubes containing 5 ml LB plus 100 μg ml^-1^ carbenicillin. Experimental controls were βLR2, a metagenomic clone containing a metallo-β-lactamase [2], and EPI300 *E*. *coli* containing pCF430. Cultures were shaken at room temperature for 48 hr. At 30 minutes, 6 hr, 24 hr, and 48 hr post-inoculation, 500 μl was removed and centrifuged. 400 μl of each supernatant was pipetted into a well in the MH agar. Plates were incubated at room temperature for 24 hr, at which time the diameter of the zone of growth inhibition was measured.

### Subcloning the genes encoding the metallopeptidase and response regulator

The full-length metallopeptidase gene alone (pCRC01), the full-length regulator gene alone (pCRC03) and the combination of both the metallopeptidase and the regulator gene (pCRC02) were subcloned in a pET28b (Novagen, Madison, WI) background using primers (Table 2) targeting *Xba*I and *Bam*HI restriction enzyme sites (pCRC01, pCRC02), or *Nco*I and *Bam*HI restriction enzyme sites (pCRC03) present in the pET28b multicloning region. A Thr to Ala mutation was introduced at Thr2 in the regulator gene coding sequence in pCRC03 to facilitate cloning using the *Nco*I restriction enzyme site.

**Table 2:** Primers (5′-3′) used for cloning and qPCR/RT-PCR.

Clone	Forward primer	Reverse primer
**Subcloning the active gene** ^a^		
pCRC01	cccctctag*a* *aggag*aacggcATGCTTCTAGGCATGCTGATGGG	cgtcggatccTTATGTAGAGCTGATGCATGACGCCTGTTCTCCG
pCRC02	cccctctag*a* *aggag*aacggcATGCTTCTAGGCATGCTGATGGG	cgtcggatccTTAGTTGTTAGACACCTCAAATCGATAGCCAATTCC
pCRC03	cagtccATGGCGAATAATGATGTGCATATTCTGGTAGTTG	cgtcggatccTTAGTTGTTAGACACCTCAAATCGATAGCCAATTCC
***uvrA* promoter**		
pCRC01UVRA, pCRC02UVRA, and pCRC03UVRA	GATCTGATATCTTTACACTTTATGCTT	CTAGATTATACGAGCCGGAAGCATAA
**qPCR/RT-PCR**		
*acrA* ^b^	CTTAGCCCTAACAGGATGTG	TTGAAATTACGCTTCAGGAT
*acrB* ^b^	CGTACACAGAAAGTGCTCAA	CGCTTCAACTTTGTTTTCTT
*acrD* ^b^	GATTATCTTAGCCGCTTCAA	CAATGGAGGCTTTAACAAAC
*mdtA*	TAACGCGCGCATGTTAGT	GCTATTCAGCACCCAGACAA
*mdtB*	AACTGCGTCGTCCGTTAG	GCGGTCGAACAGCAAATAAA
*ompF*	GGCGCAACCTACTACTTCAA	GAACCTACGCCCAGTTTGT
*ompC*	GTCCACCTACGTTGACTACAAA	GACCCAGAGCTACGATGTTATC
*micF* ^b^	TCATCATTAACTTTATTTATTACCG	GCATCCGGTTGAAATAGG
*GAPDH* ^b^	ACTTACGAGCAGATCAAAGC	AGTTTCACGAAGTTGTCGTT

^a^Capital letters indicate coding regions, underline indicates restriction enzyme sites, and italics indicate an engineered *E*. *coli* ribosomal binding site.

^b^Primers are from Viveiros et al. 2007 [23].

To construct a system in which the genes present on these constructs would be expressed in an *E*. *coli* strain in the absence of the λDE3 lysogen, a native *E*. *coli* promoter (*uvrA*) was cloned into each of these constructs using preexisting *Bgl*II and *Xba*I restriction enzyme sites from their respective pET28b multicloning regions. The cloned promoter DNA was engineered with these restriction enzyme sites, and was constructed from two synthetic oligonucleotides that were annealed and digested accordingly (Table 2). This strategy directly added the native *E*. *coli* promoter, displacing the existing T7 promoter region. The newly constructed vectors were named pCRC01UVRA, pCRC02UVRA, and pCRC03UVRA, according to the notation introduced in the above paragraph. Verification of expression was performed by SDS-PAGE analysis of the protein from EPI300 *E*. *coli* containing pCRC03UVRA as well as phenotypic assays.

### Gene expression analysis

Quantitative PCR following reverse transcription of purified *E*. *coli* RNA was used to assess expression of the *acrA*, *acrB*, *acrD*, *mdtA*, *mdtB*, *ompC*, and *ompF* genes, as well as the small RNA *micF*. EPI300 harboring pCRC03UVRA or pUVRA28b was grown in LB supplemented with kanamycin to an OD_600_ value of 0.5 and the cells were harvested by centrifugation. Total RNA was purified using the RNeasy Mini kit according to the manufacturer’s specifications (Qiagen). Purified RNA was quantified using a Take3 micro-volume plate with UV spectrophotometry (BioTek) and supplemented with the SUPERase•In RNase inhibitor (Life Technologies). Purified RNA was subjected to RNase-free DNase (Promega) treatment to eliminate any contaminating DNA. 500ng DNase-treated RNA was used for cDNA synthesis using SuperScript VILO mastermix (Invitrogen) in a 20μl reaction at 42°C for 2 h. Quantitative PCR was performed on 1μl of a 1:8 dilution of the resulting cDNA in a 10μl reaction that contained 300 nM PCR primers (Table 2) and SsoAdvanced Universal SYBR Green Supermix (Bio-Rad). The PCRs amplified a ~70–200 base region of DNA in each case (acrA 189 bp, acrB 183 bp, acrD 187 bp, mdtA 103 bp, mdtB 101 bp, ompF 92 bp, ompC 95 bp, micF 70 bp). Quantitative PCR was performed on a CFX384 real-time PCR detection system (Bio-Rad) using the following cycling parameters: one cycle at 95°C for 10 min as hot start, followed by 40 cycles of denaturation at 95°C for 30 s, annealing at 47°C (*acrA/B/D*) or 52°C (*mdtA/B*) or 55°C (*ompC/F*) or 45°C (*micF*) for 30 s, and extension at 72°C for 30 s. Control PCRs were also included using a reverse transcriptase-minus (RT-minus) template to verify that genomic DNA did not contribute to the observed results. Expression levels under each condition were normalized to the *GAPDH* housekeeping gene [23]. Melting curve analysis (65–99°C with a heating rate of 1°C per second and a continuous fluorescence measurement) was done to verify the identity of the PCR products. The 2^-ΔΔCt^ method was used to calculate fold change in gene expression [24].

## Results

### Identification of βLR16

Four metagenomic libraries from Alaskan soil DNA were selected on various β-lactam antibiotics, yielding 14 resistant clones [2]. Thirteen of the clones encoded β-lactamases, but one did not. This clone, designated βLR16, grew in the presence of carbenicillin (50 μg ml^-1^) at 24°C but not at 37°C. When resuspending cell pellets of βLR16, it was noted that the pellet was stickier and harder to resuspend than *E*. *coli* containing empty vector.

### Phenotypic characterization of βLR16

To assess the range of resistance conferred by the βLR16 clone, resistance to 15 antibiotics was tested in minimum inhibitory concentration (MIC) assays. βLR16 conferred elevated and specific resistance to carbenicillin on EPI300 *E*. *coli* (Table 3). When tested against additional β-lactams, the βLR16 clone was consistently more resistant than EPI300 *E*. *coli* containing empty vector, but the difference was never more than 2-fold.

**Table 3:** Minimum inhibitory concentration (MIC; μg ml^-1^) of β-lactams on recombinant *E*. *coli* cells harboring constructs from this work.

Construct^a^	Antibiotic^b^							
	Amo	Amp	Cfm	Cft	Cfx	Cph	Crb	Pip
βLR16 in EPI300	4	4	<0.5	1	4	4	64	4
pCF430 in EPI300	2	2	<0.5	<0.5	2	2	8	2
pCRC03UVRA in EPI300	4	4	1	1	8	4	128	4
pUVRA28b in EPI300	2	4	<0.5	<0.5	2	4	8	4
pCRC03UVRA in BL21(DE3)							8	
pUVRA28b in BL21(DE3)							2	
βLR16 in BW25113							32	
pCF430 in BW25113							8	
βLR16 in ΔompF							32	
pCF430 in ΔompF							16	
βLR16 in ΔompC							16	
pCF430 in ΔompC							2	

^a^ pCRC01UVRA showed no difference in levels of Crb resistance compared to vector alone (pUVRA28b), while pCRC02UVRA showed the same Crb resistance level as pCRC03UVRA. ΔompF and ΔompC are in the BW25113 *E*. *coli* background and are from the Keio collection of gene knockouts (see Table 1).

^b^ Antibiotic abbreviations are as follows: Amo, Amoxicillin; Amp, ampicillin; Cfm, cefamandole; Cft, ceftazidime; Cfx, cefoxitin; Cph, cephalexin; Crb, carbenicillin; Pip, piperacillin.

Resistance to carbenicillin was examined in a zone-of-inhibition assay to assess whether the mechanism of resistance conferred by βLR16 resulted from antibiotic inactivation. After 48 hours, the zone diameter resulting from the supernatant of a βLR16 culture was the same as the zone diameter from the supernatant of carbenicillin-containing media inoculated with *E*. *coli* containing empty vector (Table 4). This was in contrast to the diameter of the zone resulting from the supernatant of the 48-hour βLR2 culture (Table 4). βLR2 contains a gene encoding a metallo-β-lactamase homologue, and over time this clone inactivated the carbenicillin in the media and reduced the size of the zone of inhibition of *E*. *coli*. The zone observed with the βLR16 supernatant was not different from the negative control, suggesting that the resistance does not result from inactivation of carbenicillin.

**Table 4:** Zones of inhibition of EPI300 *E*. *coli* growth resulting from 400 μl supernatant following incubation with 100 μg ml^-1^ carbenicillin.

Plasmid^a^	zone diameter^b^			
	t_0_ ^c^	t_1_	t_2_	t_3_
pCF430	17	18	16	16
βLR2	18	16	11	10
βLR16	20	19	15	16

^a^All plasmids in EPI300 *E*. *coli*. pCF430 is empty vector, and βLR2 contains a putative metallo-β-lactamase.

^b^10 mm-diameter well.

^c^Samples taken at 30 minutes (t_0_), 6 hours (t_1_), 24 hours (t_2_), and 48 hours (t_3_) post-inoculation.

### Sequence analysis

**Fig 1:**
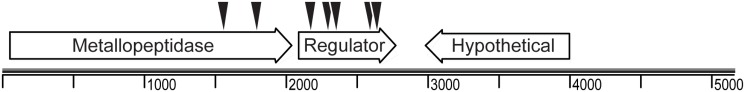
Open reading frame (ORF) map of the insert of metagenomic clone βLR16. Gray triangles designate the locations of transposon insertions that eliminated the resistance phenotype.

The DNA insert from the βLR16 metagenomic clone was 5,169 base pairs. Sequence analysis further supported the phenotypic results, showing that the metagenomic DNA insert from βLR16 did not encode a predicted β-lactamase gene (Fig. 1). *In silico* open reading frame (ORF) analysis suggested two ORFs that share homology to known genes, and loss-of-phenotype transposon mutants mapped to these two putative genes (Fig. 1). The location of the loss-of-phenotype transposon insertions suggested that either both genes were required for resistance, or that mutations in the upstream metallopeptidase gene were polar on the downstream response regulator gene (Fig. 1).

A blastP query using the predicted amino acid sequence of the βLR16 upstream gene product showed the highest identity with a hypothetical protein from the unclassified Zetaproteobacterium SCGC AB-137-C09 (WP_018282215.1), at 39% identity over 632 amino acids. This βLR16 protein showed evidence of an N-terminal urea transport membrane domain, and a peptidase family M23 domain (zinc metallopeptidase). A blastP query using the predicted amino acid sequence of the βLR16 downstream gene product showed a putative transcriptional regulator from the Betaproteobacteria *Pseudogulbenkiania ferrooxidans* (WP_008955746.1) to have the highest identity, with 56% identity over 228 amino acids. This βLR16 protein is predicted to encode an N-terminal response regulator receiver domain (Conserved Domain cd00156) and a C-terminal effector domain (Conserved Domain cd00383) [25], and can be assigned to the OmpR/PhoB family of response regulators [26]. The function of either of the proteins described above in their host organism is not known.

**Fig 2:**
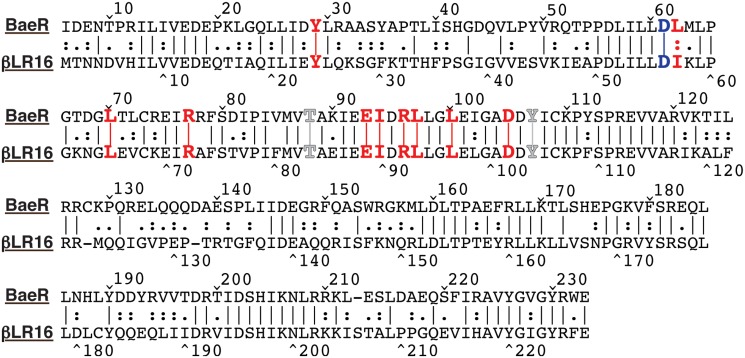
Amino acid alignment of the BaeR response regulator of *E*. *coli* and the response regulator from βLR16. Alignment was performed with MegAlign from the Lasergene software package from DNASTAR (Madison, WI) using the Lipman-Pearson alignment tool (Ktuple:2; Gap Penalty: 4; Gap Length Penalty: 12). Levels of similarity between the sequences are indicated as lines (identical) or dots (similar). The conserved aspartate residue that is phosphorylated is indicated in blue (Asp 56 in βLR16). Switch residues are indicated in gray (Thr 83 and Tyr 102 in βLR16), and amino acids from BaeR involved in domain swap interactions, and their βLR16 counterparts, are shown in red.

Considering that the response regulator gene may impact gene expression in the *E*. *coli* host, a blastP query of *E*. *coli* K-12 (substr. MG1655) was conducted to determine whether the βLR16 response regulator shows homology with a known *E*. *coli* response regulator. This search revealed that the βLR16 regulator protein sequence showed the highest identity with the BaeR response regulator protein (NP_416583.1), with 51% identity over 227 amino acids. Alignment of the βLR16 regulator protein with BaeR (Fig. 2) revealed conservation of key amino acids, including the aspartate residue (Asp 56 in βLR16) that is phosphorylated in the receiver domain, and the “switch residues” (Thr 83 and Tyr 102 in βLR16) that undergo distinct rotameric shifts upon domain activation. Further, nine of the ten amino acids involved in domain swap dimerization interactions necessary for activation in the BaeR protein are conserved in the βLR16 protein [27] (Fig. 2).

### Analysis of βLR16 subclones

To assess the potential roles of the putative metallopeptidase and response regulator genes in resistance, subclones were generated and carbenicillin resistance was assayed in an *E*. *coli* EPI300 background. Analysis of subclones harboring the metallopeptidase gene alone (pCRC01UVRA), the two genes together (pCRC02UVRA), and the response regulator gene alone (pCRC03UVRA) revealed that the response regulator gene was necessary and sufficient to confer carbenicillin resistance on the *E*. *coli* host (Table 3). No resistance was seen with the metallopeptidase alone, while the combination of the two genes yielded a resistance profile that was the same as the regulator gene alone. Based on these results, we hypothesized that the two-component regulator gene may be altering gene expression in *E*. *coli* in a way that results in elevated, specific resistance to carbenicillin.

### MIC analysis in different *E*. *coli* strains

Antibiotic susceptibility was analyzed for pUVRA28b (empty vector) and pCRC03UVRA (response regulator subclone) in *E*. *coli* strains EPI300 and in BL21(DE3) (Table 3). A four-fold increase in MIC with carbenicillin can be seen with empty vector between BL21(DE3) and EPI300, presumably due to genomic differences between the K-12-derived (EPI300) and B-derived (BL21(DE3)) strains. The increase in carbenicillin resistance conferred by the response regulator subclone is significantly greater in the EPI300 background when compared to the same vector in the BL21(DE3) background; nonetheless, some increase in resistance is noted in BL21(DE3). Expression of the regulator gene from pCRC03UVRA in EPI300 did not increase resistance to certain outer membrane-permeating antibiotics such as erythromycin, fusidic acid, and gentamicin (data not shown). Owing to the differences in porin content and regulation between the two strains, we reasoned that variations in expression of the OmpC or OmpF porin could result in the observed carbenicillin resistance phenotype. We therefore employed single gene, in-frame deletions of the *ompF* and *ompC* genes (Table 1) for two purposes. Assessing the relative carbenicillin resistances of the deletion strains compared to the wild type strain would allow us to determine whether regulation of these genes may be involved in carbenicillin resistance, and to determine whether the βLR16 regulator gene impacts resistance in the absence of either of the porin genes. The kanamycin resistance cassette insertions in the ΔompC and ΔompF strains (Table 1) precluded use of the pCRC03UVRA subclone in these strains, owing to the kanamycin resistance selectable marker in the vector. Comparing the empty vector (pCF430) MIC values between the K-12-derived strain (BW25113, the progenitor for the Keio knockout collection [28]) and the two porin knockout strains, the data suggested that elimination of the OmpF porin increases carbenicillin resistance while elimination of the OmpC porin renders *E*. *coli* more sensitive to carbenicillin. The βLR16 clone enhances carbenicillin resistance in each of the strains, with the greatest difference between empty vector and βLR16 resistance in the ΔompC strain. This is consistent with downregulation of *ompF* expression as a means of carbenicillin resistance, but the enhanced resistance conferred by βLR16 in the ΔompF strain suggests the involvement of additional mechanisms.

### Gene expression analysis

The expression of genes involved in pumping β-lactams (*acrA*, *acrB*, *acrD*, *mdtA*, *mdtB*) as well as genes involved in β-lactam outer membrane permeability (*ompC*, and *ompF* porin genes, and the *micF* small RNA) was analyzed in EPI300 grown with the regulator subclone (pCRC03UVRA) as compared to EPI300 grown with the empty vector (pUVRA28B) (Table 5). The *acrA* and *acrB* genes showed less than twofold change in expression under these two conditions, while the *acrD* gene was upregulated over 10 fold in the presence of the response regulator. The *mdtA* and *mdtB* genes both exhibited >10-fold increases in expression in the presence of the response regulator gene. Each of the three “permeability” genes (*ompC*, *ompF*, *micF*) was downregulated somewhat in the presence of the regulator subclone, but the impact was most noteworthy in the case of the *ompF* gene, where a >20-fold decrease in expression was seen. The *ompC* gene was downregulated ~2-fold, while the *micF* small RNA exhibited less than 2-fold change in expression.

**Table 5:** Fold change in gene expression of select *E*. *coli* genes in the presence of the regulator-containing vector (pCRC03UVRA) versus empty vector (pUVRA28b).

Gene	Fold change ± standard deviation
*acrA*	0.67 ± 0.09
*acrB*	0.51 ± 0.11
*acrD*	11.51 ± 3.25
*mdtA*	11.93 ± 2.03
*mdtB*	13.30 ± 1.26
*micF*	0.69 ± 0.16
*ompC*	0.45 ± 0.07
*ompF*	0.05 ± 0.01

## Discussion

The structural and mechanistic similarities between metallopeptidase enzymes and metallo-β-lactamase enzymes led us to first postulate that the putative metallopeptidase gene from βLR16 was responsible for resistance. However, the phenotypic analyses suggested that carbenicillin resistance conferred by βLR16 does not result in inactivation of the β-lactam antibiotic. Additionally, a subclone of the metallopeptidase did not increase carbenicillin resistance in *E*. *coli* EPI300, while a subclone of the response regulator gene increased resistance markedly, leading us to conclude that the response regulator was necessary and sufficient for β-lactam resistance. Considering the nature of the resistance gene, we hypothesized that the response regulator altered expression of one or more native *E*. *coli* genes that would allow for increased, specific tolerance to the β-lactam carbenicillin

Previous work has established that spontaneous mutants showing a phenotype similar to what we observed (temperature-dependent carbenicillin resistance) map to the *ompF* gene in *E*. *coli* and other Gram negative organisms [29,30]. While porin specificity is dictated by the size, charge, and hydrophobicity of the substrate [31], OmpF is thought to preferentially allow influx of anionic compounds, while OmpC is more cation selective [31]. It is noteworthy that carbenicillin is a dianionic molecule, while the other β-lactams tested here are monoanionic or zwitterionic. This may explain the apparent selectivity of the OmpF porin for carbenicillin, as opposed to structurally related monoanionic or zwitterionic β-lactams. Nonetheless, both porins are known to be involved in antibiotic influx [30].

The resistance phenotype analyses showed that the *E*. *coli* BW25113 *ompF* knockout (Δ*ompF*) displayed slightly elevated resistance to carbenicillin compared to the wild type strain. In contrast, the *ompC* knockout (Δ*ompC*) was more susceptible. This is consistent with previous work showing that the OmpF porin is the primary porin through which carbenicillin enters the *E*. *coli* periplasm [29]. Considering that either porin is likely overrepresented in the absence of the other, a proportionally larger quantity of OmpF (in the Δ*ompC* strain) would mean a higher influx of carbenicillin. The qRT-PCR data were consistent with a role for the response regulator in decreasing expression of the *ompF* gene—in a *micF*-independent manner—in *E*. *coli*.

The 2-fold increase in resistance in the Δ*ompF* strain suggested that the regulator impacts more than just expression of the *ompF* gene, so genes encoding efflux pumps were investigated. Previous work indicates that the AcrAB-TolC pump system of *E*. *coli* has very broad substrate specificity and can enhance resistance to a wide variety of antibiotics, detergents, and solvents [32]. This pump is also proposed to be the primary means by which β-lactam antibiotics are pumped from the cytoplasm or periplasm into the extracellular space [33]. Indeed, both deletion and overexpression studies of the *acrAB* genes in *E*. *coli* show that these genes confer “across the board” resistance to β-lactams [34]. In contrast, overexpression of the *mdtABC* genes and *acrD* gene show enhanced, yet specific, carbenicillin resistance [32]. These observations are consistent with our gene expression analysis, which showed negligible changes in expression to the *acrA* and *acrB* genes in the presence of the βLR16 response regulator gene, yet increases in expression of both the *acrD* gene as well as the *mdtA* and *mdtB* genes. The βLR16 response regulator gene, therefore, is responsible for both repressing porin expression and activating multidrug efflux pump expression.

Regulation of the *mdtABC* genes and the *acrD* gene by the same response regulator is not without precedent. The BaeSR two-component system of *E*. *coli* is shown to activate expression of both the *mdtABC* genes and the *acrD* gene [35,36]. Overall similarities (Fig. 2) between the βLR16 response regulator and the BaeR response regulator suggest that they may act in a similar fashion to enhance antibiotic resistance. Conversely, the βLR16 response regulator may act as a repressor in a manner that indirectly impacts gene expression to give the observed results.

This study introduces a number of concepts that should be further explored, including alternative mechanisms by which gene regulation can impact antibiotic tolerance, co-regulation of antibiotic influx and efflux genes, and the genetics and biochemistry of modular response regulators in alternative host organisms. We also propose that further explorations of the soil metagenome using functional screening and selection strategies will be an outstanding method for advancing discovery in the broader biology of microorganisms.
